# Abundance and Temperature Dependency of Protein-Protein Interaction Revealed by Interface Structure Analysis and Stability Evolution

**DOI:** 10.1038/srep26737

**Published:** 2016-05-25

**Authors:** Yi-Ming He, Bin-Guang Ma

**Affiliations:** 1 College of Life Science and Technology, Huazhong Agricultural University, Wuhan 430070, China; 2 Hubei Key Laboratory of Agricultural Bioinformatics, College of Informatics, Huazhong Agricultural University, Wuhan 430070, China

## Abstract

Protein complexes are major forms of protein-protein interactions and implement essential biological functions. The subunit interface in a protein complex is related to its thermostability. Though the roles of interface properties in thermal adaptation have been investigated for protein complexes, the relationship between the interface size and the expression level of the subunits remains unknown. In the present work, we studied this relationship and found a positive correlation in thermophiles rather than mesophiles. Moreover, we found that the protein interaction strength in complexes is not only temperature-dependent but also abundance-dependent. The underlying mechanism for the observed correlation was explored by simulating the evolution of protein interface stability, which highlights the avoidance of misinteraction. Our findings make more complete the picture of the mechanisms for protein complex thermal adaptation and provide new insights into the principles of protein-protein interactions.

Proteins are essential cellular components and the major executors for biological functions. Thousands of biochemical reactions in a cell are carried out by protein molecules and their interactions. A large part of proteins function in the form of complexes and protein complexes have quaternary structures maintained mainly by weak interactions such as hydrogen bonds, salt bridges, Van der Waals and hydrophobic forces^1^. Keeping thermostability for protein complexes in their native conformations is a key demand for their proper functions^2^. The maintenance of the stability of the protein complexes is particularly important for thermophiles with Optimal Growth Temperature higher than 50 Celsius degrees (OGT ≥50 °C) due to their elevated habitat temperatures. Previous studies showed that the characteristics of the subunit interface are major contributors to the temperature adaptation of protein complexes^3^,^4^. Our previous work has suggested that the interface of a protein complex is a distinct attribute reflecting the OGT of its host organism^5^. We found that the hydrophobicity and the pairing patterns of amino acids in the interfaces of protein complexes in thermophiles are significantly different from those in mesophiles (25 °C ≤OGT <50 °C); for example, with the increase of temperature, there will be higher hydrophobicity and more regular paring patterns of the charged amino acids in thermophilic protein complexes than their mesophilic counterparts^5^.

Due to the fact that proteins are products of expressed genes, gene expression level determines protein abundance. In a previous work, it was found that highly expressed proteins may be more thermostable than lowly expressed ones due to their unique sequence compositions^6^,^7^. However, whether the relationship between proteins’ thermostability and their abundance holds for protein complexes remains unknown. Since the thermostability of a protein complex is reflected in its subunit interface^3^,^4^, what is the connection between the interface property and the protein expression level? Furthermore, protein complexes are concrete embodiments of protein interactions and thus the discovery of this relationship is also helpful to understanding the molecular mechanisms of protein interaction. In this work, we tried to explore the relationship between the interface size and the expression level for protein complexes based on the 3D structures compiled from the PDB database^8^ and the expression levels taken from the GEO database^9^. We calculated the inter-chain interface size for the protein complexes from two thermophiles and three mesophiles. By comparison, we found that highly expressed protein complexes incline to have larger interfaces than lowly expressed ones in thermophiles while this trait is not manifested in mesophiles. Since the inter-chain interface of a protein complex is related to its thermostability and the interaction of its subunits^10^,^11^, the observed trend that highly expressed protein complexes have larger subunit interfaces revealed a positive correlation between the thermostability of protein complexes and their abundance. To seek the underlying mechanism of this correlation, we simulated the evolution of protein interface stability by using lattice models and found differences in evolutionary speed and energy change between high and normal temperature conditions, which highlighted a higher selection pressure for avoiding misinteractions between highly expressed proteins. Our results provided valuable information for understanding the molecular mechanisms that govern protein-protein interaction.

## Results

The structures for the protein complexes in two thermophilic bacteria and three mesophilic bacteria (model organisms) were collected from the PDB database and their corresponding genes were identified and downloaded from the NCBI RefSeq database^12^. By characterizing the sizes of the contacting interfaces between the subunits (chains) in the protein complexes and expression levels for these protein chains, the relationship between the interface sizes and the expression levels of the protein complexes was examined (Fig. S1). The results showed that highly expressed proteins have larger interfaces in thermophilic bacteria and this trait is lacking or less pronounced in mesophilic bacteria. To seek the underlying mechanisms for the observed correlation, we simulated the evolution of protein interface stability by using lattice models and revealed the dependency of protein-protein interaction on both temperature and protein abundance.

### The distribution of interface size and expression level for the protein complexes

The basic information for the two thermophilic bacteria and three mesophilic bacteria (model organisms) was presented in Table 1. Firstly, we checked the distribution of the protein subunit interface size and expression level for the studied complexes in the five organisms and found that they are not exactly in normal distributions (Fig. S2). The basic statistics of the chain length, interface size measured by contacting residue pairs (N_aa_), interface size measured by NACCESS area (A_naccess_), expression level measured by CAI value and expression level taken from GEO database for the protein complexes were listed in Table 2. It seems no universal trend of variation exits between thermophilic and mesophilic organisms and Wilcoxon statistical tests showed that there is no significant difference of protein chain length and interface size between thermophilic and mesophilic protein complexes, while the expression levels are not directly comparable between species because they are relative values among genes inside one species.

### Highly expressed proteins have larger interfaces in thermophilic protein complexes

**Figure 1:**
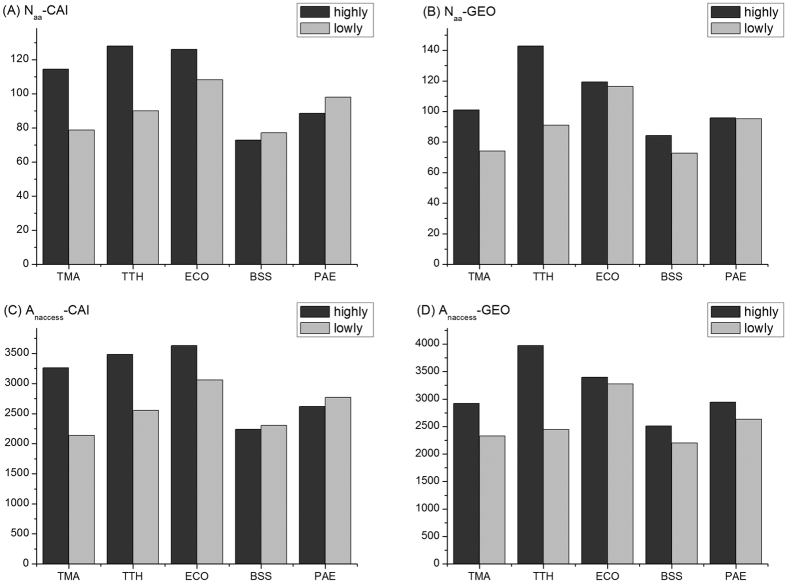
The comparison of interface size between highly and lowly expressed protein complexes. (**A**) The interface size is measured by the number of contacting residues and the expression level is measured by CAI values (N_aa_-CAI); (**B**) the interface size is measured by the number of contacting residues and the expression level is taken from GEO database (Naa-GEO); (**C**) the interface size is measured by the area (in the unit of Å^2^) calculated by NACCESS and the expression level is measured by CAI values (A_naccess_-CAI); (**D**) the interface size is measured by the area (in the unit of Å^2^) calculated by NACCESS and the expression level is taken from GEO database (A_naccess_-GEO). All the figures consistently show that highly expressed protein complexes have larger subunit interfaces than lowly expressed ones in thermophiles (TMA, TTH) while this trait is not pronounced in mesophiles (ECO, BSS, PAE).

The expression levels of the studied protein complexes were measured at transcription and translation level by GEO experimental data and the calculated CAI values, respectively. The protein chains with top 30% expression levels were considered as (relatively) highly expressed and the other 70% lowly expressed. We also checked other partition schemes, for example, 20% vs. 80%, 40% vs. 60%, and half-half, and the results are qualitatively similar (data not shown). Then the average interface sizes for the highly and lowly expressed proteins were compared and it was found that the highly expressed proteins have larger interfaces than lowly expressed proteins (Fig. 1), consistently indicated by the combinations of the two measures for interface size (namely, the number of contacting residue pairs N_aa_ and the NACCESS interface area A_naccess_ and the two measures for expression level: CAI and GEO).

The significance of the above observation is supported by statistical tests. As shown in Table 2, the interface sizes are significantly larger in highly expressed protein complexes in thermophiles; for example, for the N_aa_-CAI combination (Fig. 1A), the interface sizes for highly expressed protein complexes are 45% and 42% percent larger than the lowly expressed protein complexes in thermophiles TMA and TTH, respectively, and the comparison by using Wilcoxon tests indicates that the difference is significant with *p* = 0.003 for TMA and *p* = 0.008 for TTH, respectively (Supplementary Table S1); similar results are true for other combinations of N_aa_-GEO, A_naccess_-CAI and A_naccess_-GEO (Fig. 1B–D) with significant differences indicated by corresponding *p*-values at a level of not larger than 0.05 (Supplementary Table S1).

### This trait is less pronounced in mesophilic protein complexes

To check if the trait that highly expressed protein complexes have larger subunit interfaces is universal or just holds for thermophiles, the protein complexes for three mesophilic model organisms (Table 1) were also collected and the same analysis was performed on these data for comparison. As shown (Fig. 1 and Supplementary Table S1), the differences of interface size between the highly expressed protein complexes and the lowly expressed ones are much smaller in the three mesophiles compared with those in thermophiles. The statistical tests showed almost no significant differences at a level of *p*-value = 0.05 except for some combinations (N_aa_-CAI and A_naccess_-CAI) for *E. coli* (Fig. 1A,C and Supplementary Table S1) and the significance of these differences are mainly owing to the extremely large data set for this extensively used model organism, with more than 1000 protein complex structures in *E. coli* versus about 100 ones in other organisms (Table 1). On the other hand, for the combinations (N_aa_-CAI and A_naccess_-CAI) in mesophilic organisms BSS and PAE, the interfaces of highly expressed protein complexes are even smaller than those of the lowly expressed ones (Supplementary Table S1 and Fig. 1A,C). Therefore, we concluded that the protein complexes with higher expression levels have larger interfaces mainly holds for thermophiles, while for mesophiles, this trait is absent or relatively weak.

### This trait is correlated with binding energy and chemical bonds of contacting residue pairs

What does this trait mean in terms of physicochemical property? We calculated the binding energy and chemical bonds based on the contacting amino acid pairs in the interface and made comparison between highly (top 30%) and lowly (the other 70%) expressed protein complexes measured by GEO expression data. We found that the highly expressed protein complexes in thermophilic organisms have significantly greater (in absolute value) average binding energy than lowly expressed ones (Table 3), suggesting that the highly expressed protein complexes in thermophilic organisms are more thermostable in quaternary structure. In contrast, the average binding energy of the interfaces in mesophilic protein complexes shows inconsistent or opposite differences between highly and lowly expressed ones (Table 3). Similarly, the numbers of hydrogen bonds and salt bridges in the interfaces of the highly expressed protein complexes are significantly greater than those of lowly expressed ones in thermophiles while such a trend is lacking or less pronounced in mesophiles (Table 3). Therefore, the larger interface in the highly expressed thermophilic protein complex is an indicator of the higher thermostability embodied in greater binding energy and more chemical bonds in the inter-chain interface composed of contacting amino acids.

### Simulation shows different evolutionary speed of interface stability at high and normal temperature conditions

We simulated the evolution of protein interface stability by using lattice models for the high temperature (350K) and normal temperature (310K) conditions, respectively (Supplementary Method). The evolutionary speed is represented by slope of the linear fitting for the correlation between the variation of binding energy of protein interaction with the generation number during evolution. We found that the protein stability increases with evolution for both the two temperature conditions, showing that stability has evolutionary benefits (Supplementary Method). When the proteins were classified into two groups according to the expression level, we found that at high temperature condition the evolution speed of interface stability of the highly expressed proteins is higher than that of the lowly expressed proteins, while at the normal temperature condition the evolution speed of the highly expressed proteins is close to or even lower than the lowly expressed ones (Table 4, Supplementary Method). We also simulated the evolution of interface stability at a middle level temperature of 330K and obtained results similar to the situation at high temperature condition (data not shown). Considering the fact that the evolution speed of highly expressed proteins is lower than the lowly expressed ones^13^, the higher evolutionary speed of the highly expressed protein complexes is not trivial because the protein interface stability has to be preferentially optimized by sufficient using of the very limited sequence mutations.

### Simulation shows different energy change of protein interface at high and normal temperature conditions

**Figure 2:**
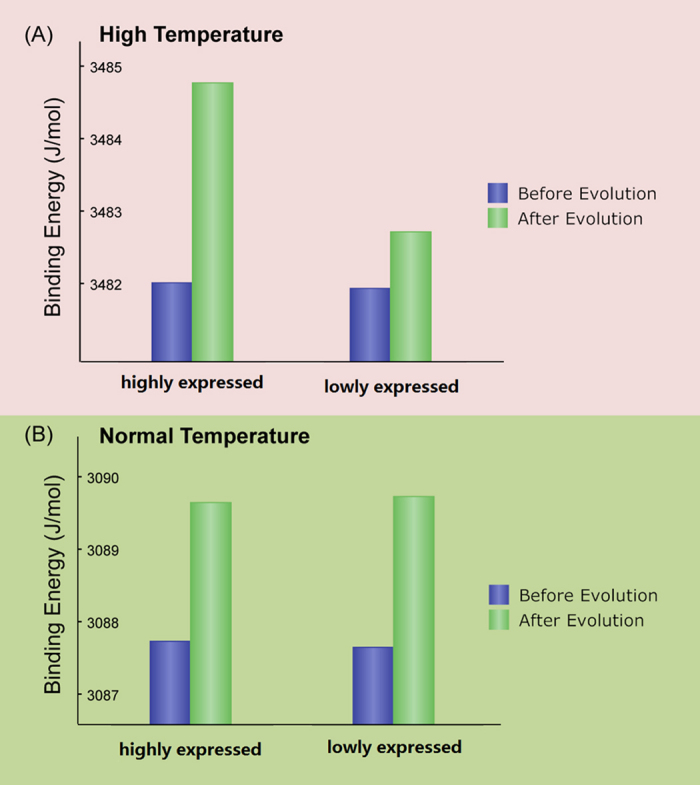
The binding energy change for the highly and lowly expressed protein complexes before and after evolution simulated at high and normal temperature conditions. (**A**) At high temperature condition, the energy change of the highly expressed proteins is obviously larger than that of the lowly expressed ones; (**B**) at the normal temperature condition, there is no difference of the energy change for the highly and lowly expressed proteins.

Indeed, the multimerization of proteins in living cells proceeds in an environment of thermal noise. To maintain the robustness of protein interaction, there should be an energy gap between the native and the mis-interacted conformations of protein complexes and this energy gap could be optimized by evolution. As shown in Fig. 2, the average binding energy between the interacting protein pairs was optimized (increased in the absolute value) during evolution for both the highly and lowly expressed protein complexes at the two temperature conditions. However, the energy change is different: at the high temperature condition, the binding energy for the highly expressed protein complexes has obviously larger change after evolution than the lowly expressed proteins, while at the normal temperature condition, the binding energy change after evolution has no obvious difference between the highly and lowly expressed protein complexes. These results indicate that the protein interface stability has been preferentially optimized to avoid misinteraction for the highly expressed protein complexes at the high temperature condition that corresponds to the higher evolutionary pressure in thermophiles, while the protein interface stability has no preferential optimization for the highly expressed protein complexes at the normal temperature condition that corresponds to the lower evolutionary pressure in mesophiles.

## Discussion

**Figure 3:**
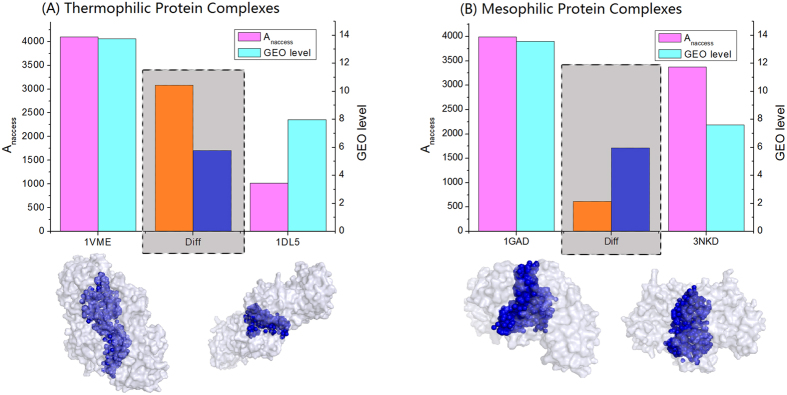
A schematic illustration for the differences between thermophilic and mesophilic protein complexes. (**A**) Two example protein complex structures (PDB code: 1VME and 1DL5) with high and low expression level respectively from thermophile *Thermotoga maritima* MSB8; (**B**) two example protein complex structures (PDB code: 1GAD and 3NKD) with high and low expression level respectively from mesophile *Escherichia coli* K-12 MG1655. The left and right y-axis is NACCESS area (in the unit of Å^2^) and GEO data to indicate the subunit interface size and expression level, respectively. The pictures at the bottom of each sub-graph are the PDB structures rendered by PyMol to illustrate the subunit interface (in blue color) and the overall surface (in gray color). The “Diff” columns in the middle (colored by orange and navy with gray background) show the differences of interface size and expression level between highly and lowly expressed protein complexes from thermophile and mesophile, respectively. The “Diff” columns in sub-graphs (**A**) and (**B**) schematically show the opposite trend that for thermophilic protein complexes, a small change of expression level corresponds to a big change of interface size while for mesophilic complexes, a big change of expression level corresponds to a small change of interface size, meaning that significant positive correlation between interface size and expression level exists for thermophilic protein complexes while not for mesophilic complexes.

We have studied the interface size in the protein complexes from thermophiles and mesophiles and the relationship between the interface size and the protein expression level. It was found that highly expressed proteins incline to have larger subunit interfaces than lowly expressed ones and this trait is significant for thermophiles and weak or absent for mesophiles (see Fig. 3 for a schematic illustration). The results showed an undiscovered relationship between interface size and subunit abundance in the protein complexes, making more complete the picture of thermal adaptation.

The finding that highly expressed protein complexes have larger subunit interfaces in thermophiles (Fig. 3A) rather than in mesophiles (Fig. 3B) gives new insights into the mechanisms of protein interaction. Our results showed that protein interaction strength, indicated by interface size (Supplementary Table S1), binding energy and chemical bonds (Table 3), is not only temperature-dependent but also protein-abundance-dependent. As previously noticed, the strength and forms of protein-protein interactions are mainly determined by the sequence or structure properties of the interacting partners^14^. There was also finding that protein interacting partners have a higher possibility of co-expression^15^. However, the relationship between the protein interaction strength (reflected by the subunit interface size) and the expression levels of the corresponding subunits remains unknown, particularly in the context of thermophilic adaption. Our finding of the positive correlation between subunit interface size and expression level filled this knowledge gap and particularly disclosed the abundance-dependency of protein complex formation and protein-protein interaction in thermophilic adaptation.

The positive correlation of subunit interface size and the subunit expression level in thermophiles rather than mesophiles might be a result of the evolution pressure for keeping thermostability of protein complex while avoiding misinteraction between subunits. As reported recently, the avoidance of protein misinteraction could significantly affect the evolution rate of highly expressed proteins^16^ and the propensity of non-specific interactions is inversely correlated with the protein abundance^17^. Larger and more specific interfaces^5^ for more abundant protein complexes in thermophiles would definitely benefit the avoidance of misinteraction of their subunits under hot environment conditions, while in mesophiles such a selection pressure is not as strong as in thermophiles due to a moderate environment temperature that results in the absent or less pronounced positive correlation between interface size and subunit expression level. Simulations revealed that protein interfaces have higher evolutionary speed and larger binding energy change for the highly expressed protein complexes at high temperature condition, which effectively confirmed the above conclusion.

## Materials and Methods

### Collection of the structures for protein complexes

The PDB database was checked recently to get the protein complexes in thermophiles and the structures were selected according to the following criteria: (1) pure protein complexes with chain number ≥2; (2) source from prokaryotes (bacteria or archaea); (3) by X-ray experiment method; (4) resolution better than 2 Å; (5) redundancy removed at sequence identity 90%. After selection, only two thermophilic organisms (Thermotoga maritima MSB8 and Thermus thermophilus HB8) have protein complex numbers larger than 100 and were used in the present analysis. For comparison, the protein complexes from three mesophilic model organisms (*Escherichia coli* K-12 MG1655, *Bacillus subtilis subsp.* subtilis str. 16, *Pseudomonas aeruginosa* PAO1) were downloaded as well following the same selection criteria. The downloaded structures were filtered to remove possible errors in the PDB files. The basic information for the finally used dataset was presented in Table 1.

### Collection of the corresponding genes (DNA sequences) for the protein complexes

The DNA sequences for the genes of the studied organisms were downloaded from the NCBI RefSeq database^12^. The protein sequences were extracted from the PDB files and the correspondence between the protein sequences and their encoding genes (DNA sequences) was identified by using the ‘tblastn’ program in the BLAST sequence alignment package with E-value <10^−7^ and other parameters as defaults. For alignment output, the DNA sequence with the highest score (best hit) was assigned to each protein sequence.

### Identification of the interfaces in protein complexes

The interfaces between protein chains in the protein complexes were identified in two ways. One way is by the contacting amino acids: if two residues in two opposite chains have a distance ≤5 Å, they are considered contacting; the interface between two chains in a complex is comprised by the contacting residues; the size of the interface is defined in this case as the number of the contacting residues (N_aa_); the interface with more than 10 contacting residues (namely, size ≥10) were considered effective and used in the analysis. The number of contacting amino acid pairs was calculated based on the BioPDB model in the Biopython package^18^. The other way is by using the NACCESS program^19^: the solvent accessible area for each protein complex was calculated; and after that, each protein complex was split into separate chains and the solvent accessible area for each chain was calculated as well; using the sum of the solvent accessible area for two chains in separate state minus the overall solvent accessible area of the two chains in associated state to represent the area of the interface part (A_naccess_); if the area of the interface is larger than 50 Å^2^, it was regarded as an effective interface. The average interface size/area for all the effective interfaces in each complex was used in comparison.

### Measurement of expression levels

The protein expression levels were measured at two layers: the transcription level and the translation level. The transcription levels for the studied genes were taken from Gene Expression Omnibus (GEO) database^20^. The latest microarray data measured for the wild-type of each organism were used in the analysis. Due to the lack of experimentally characterized protein abundance levels in thermophiles, the translation levels for the studied proteins were measured in the Codon Adaptation Index (CAI) values^21^. CAI is a widely adopted indicator of protein expression level and has been confirmed by experiments repeatedly^22^,^23^. Following the standard procedure, by taking the ribosome genes as the reference, the ‘cusp’ and ‘cai’ programs in the EMBOSS software package were used for the calculation of the reference codon usage table and the CAI values for the studied genes, respectively, in each of the five studied organisms. The protein complexes with top 30% expression levels were taken as (relatively) highly expressed proteins while the others are lowly expressed proteins.

### Analysis of the binding energy and chemical bonds of protein complex interfaces

The interface between two chains in a protein complex has its special contacting amino acid pairs. We calculated the binding energy and chemical bonds of an interface based on the amino acid contacting pattern. Each contacting amino acid pair has a particular binding energy which is measured by the MJ matrix^24^. The binding energy of an interface is defined as the sum of the binding energy of all contacting amino acid pairs. The average binding energy was calculated over all the effective interfaces in a protein complex and was compared between highly and lowly expressed protein complexes. Two types of chemical bonds were considered for an interface: hydrogen bonds and salt bridges. We employed the PDBePISA server^25^ (https://www.ebi.ac.uk/pdbe/pisa/) to count the numbers of these two types of bonds in each interface and compared the average bond number over all effective interfaces in protein complexes between highly and lowly expressed proteins.

### Simulation for the evolution of protein interface stability

The evolution of protein interface stability was simulated by using lattice models. In the simulation, 100 virtual cells were generated as a population for each generation during the evolution and within each cell 60 proteins were constructed by using a 3*3*3 lattice model. Random amino acid mutations and genetic drifts were considered during the evolution process. The conformations of interacting proteins pairs were generated for each cell in each generation and the interacting interface of a protein pair were represented by 9 contacting residue pairs on the two sides of two lattices in a particular orientation. The binding energy between two proteins (a protein pair) was calculated based on contacting potentials between amino acids. The fitness of a cell was defined as the sum of the abundance of interacting proteins within it in each generation. The detailed procedure for the simulation was presented in the Supplementary Method.

## Additional Information

**How to cite this article**: He, Y.-M. and Ma, B.-G. Abundance and Temperature Dependency of Protein-Protein Interaction Revealed by Interface Structure Analysis and Stability Evolution. *Sci. Rep.* **6**, 26737; doi: 10.1038/srep26737 (2016).

## Supplementary Material

Supplementary Information

## Figures and Tables

**Table 1:** The basic information for the studied organisms.

Species	Abbreviation	OGT (°C)	PDB No.^a^	Gene No.^b^	GEO No.^c^
*Thermotoga maritima* MSB8	TMA	80	165	105	106
*Thermus thermophilus* HB8	TTH	75	126	107	105
*Escherichia coli* K-12 MG1655	ECO	37	1025	664	614
*Bacillus subtilis* subsp. subtilis str. 16	BSS	25–35	171	123	121
*Pseudomonas aeruginosa* PAO1	PAE	25–30	187	140	136

^a^“PDB No.” is the number of protein complex structures taken from PDB database.

^b^“Gen No.” is the number of the genes corresponding to the chains in the protein complexes.

^c^“GEO No.” is the number of the genes having GEO records.

**Table 2:** The basic statistics for chain length, interface size and expression level of the protein complexes from the five organisms.

Species	Chain Length	N_aa_	A_naccess_ (Å^2^)	CAI level	GEO level
TMA	275.6 ± 143.6	89.77 ± 63.33	2495 ± 1694	0.7229 ± 0.0361	10.87 ± 1.538
TTH	253.6 ± 117.5	103.3 ± 76.75	2887 ± 1915	0.7228 ± 0.0681	9.534 ± 1.203
ECO	297.1 ± 162.8	117.2 ± 96.03	3314 ± 2549	0.4873 ± 0.1170	9.907 ± 2.000
BSS	206.1 ± 103.0	76.07 ± 56.38	2289 ± 1553	0.4950 ± 0.0712	11.23 ± 2.512
PAE	217.2 ± 99.26	95.35 ± 67.63	2726 ± 1833	0.6115 ± 0.0929	7.462 ± 2.311

**Table 3:** Comparison of the interface chemical properties between highly and lowly expressed protein complexes in the five studied organisms^a^.

Chemical Property		TMA	TTH	ECO	BSS	PAE
Binding Energy (J/mol)	Highly	−369.756	−400.908	−238.572	−403.014	−273.779
	Lowly	−265.150	−301.644	−251.467	−356.307	−315.046
	Diff	−104.606	−99.264	12.895	−46.707	41.267
	*p*-value	0.011	0.031	0.633	0.037	0.844
Hydrogen Bonds	Highly	17.986	28.096	24.958	12.541	18.357
	Lowly	14.808	15.357	21.088	14.278	18.865
	Diff	3.178	12.739	3.870	−1.736	−0.508
	*p*-value	0.015	7.420E-4	0.012	0.260	0.929
Salt bridges	Highly	6.907	13.748	8.278	3.354	5.942
	Lowly	5.745	6.071	6.185	3.741	7.276
	Diff	1.162	7.676	2.093	−0.387	−1.334
	*p*-value	0.021	0.015	0.010	0.811	0.940

^a^The compared interface chemical properties are “Binding Energy”, “average number of Hydrogen Bonds” and “average number of Salt Bridges”. “Diff” denotes the difference which is defined as: chemical property value of highly expressed – chemical property value of lowly expressed; *p*-value is obtained by Wilcoxon test. See Table 1 for the abbreviations of the five species.

**Table 4:** The linear fitting for the correlation between the binding energy and generation number during the evolution of interface stability (see also Fig. M5 and Fig. M6 in the Supplementary Method).

Temperature	Expression	Slope	Standard Error	R-square	p-value
High	Highly	3.91E-04	5.21E-06	0.92	<1E-16
	Lowly	2.56E-04	5.98E-06	0.79	<1E-16
Normal	Highly	3.32E-04	7.85E-06	0.75	<1E-16
	Lowly	3.42E-04	5.95E-06	0.85	<1E-16
