# Supplementary material for: Abundance and Temperature Dependency of Protein-Protein Interaction Revealed by Interface Structure Analysis and Stability Evolution
Source: Sci Rep. 2016 May 25;6:26737. doi: 10.1038/srep26737 (PMC4879665; doi:10.1038/srep26737)
Supplement: Supplementary Information [file srep26737-s1.pdf]

Abundance and Temperature Dependency of Protein-Protein Interaction Revealed by Interface Structure Analysis and Stability Evolution

Yi-Ming He [a], Bin-Guang Ma [b,*]

[a] College of Life Science and Technology, Huazhong Agricultural University, Wuhan 430070, China
[b] Hubei Key Laboratory of Agricultural Bioinformatics, College of Informatics, Huazhong Agricultural University, Wuhan 430070, China

* Corresponding author. Fax: +86 2787280877.
E-mail address: mbg@mail.hzau.edu.cn (Bin-Guang Ma)

Supplementary Contents:

Supplementary Table S1.

Supplementary Figures S1, S2.

Supplementary Method.

Supplementary Information

Supplementary Table S1. Comparison of the interface size between highly and lowly expressed protein complexes in the five studied organisms

Species	Interface Size (Highly Expressed)	Interface Size (Lowly Expressed)	Difference in Percentage [a]	*p*-value for Wilcoxon test
Interface size measured by N_{aa} and expression level measured by CAI				
TMA	114.47	78.74	45.38%	0.0033
TTH	128.06	89.97	42.34%	0.0088
ECO	126.13	108.166	16.61%	0.0115
BSS	72.92	77.01	-5.31%	0.6441
PAE	88.64	98.05	-9.60%	0.7722
Interface size measured by N_{aa} and expression level from GEO				
TMA	101	74	36.49%	0.0200
TTH	142.875	91.05	56.92%	4.05E-4
ECO	119.32	116.29	2.61%	0.3600
BSS	84.29	72.72	15.91%	0.1540
PAE	95.78	95.17	0.64%	0.4807
Interface size measured by $A_{naccess}$ and expression level measured by CAI				
TMA	3262.11	2140.53	52.40%	6.8E-04
TTH	3482.59	2554.92	36.31%	0.0105
ECO	3631.2	3060.14	18.66%	0.0032
BSS	2240	2304.91	-2.82%	0.5841
PAE	2619.9	2771.6	-5.47%	0.6708
Interface size measured by $A_{naccess}$ and expression level from GEO				
TMA	2918.70	2325.53	25.51%	0.0540
TTH	3971.82	2445.45	62.42%	7.4E-05
ECO	3398.51	3277.15	3.70%	0.2900
BSS	2513.84	2197.62	14.39%	0.1560
PAE	2942.26	2632.33	11.77%	0.1837

[a] The difference in percentage is defined as (interface size of highly expressed - interface size of lowly expressed) / (interface size of lowly expressed) * 100%.

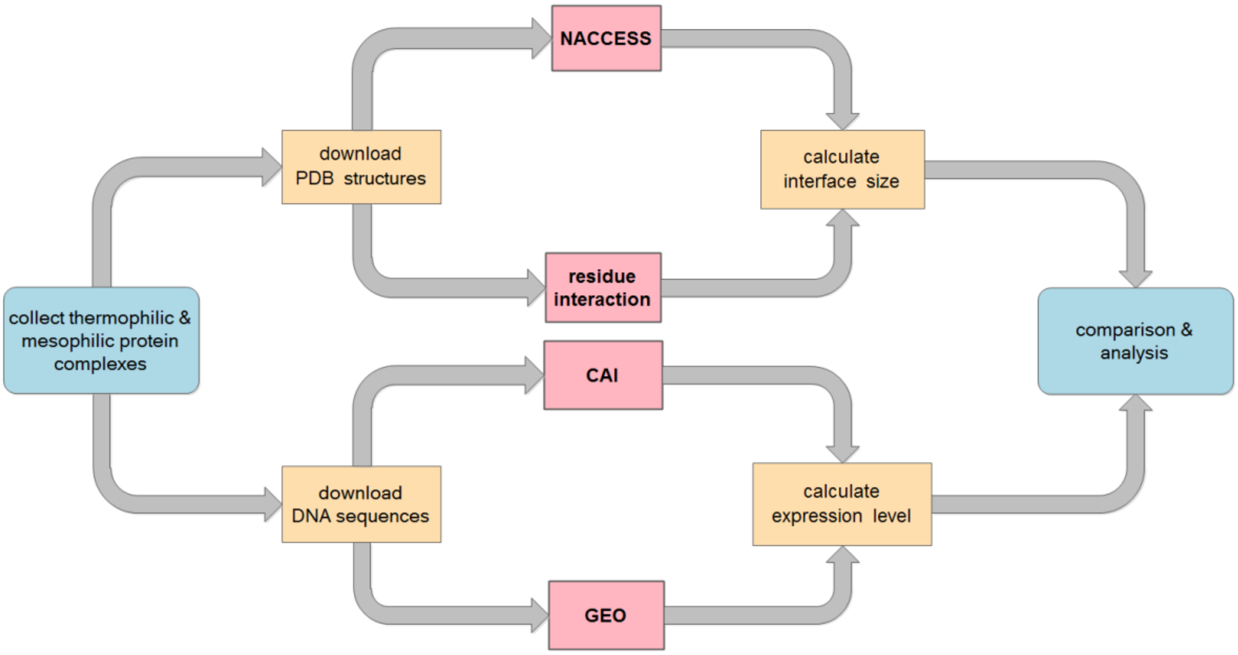

Figure S1. The flowchart for the data collection and calculation.

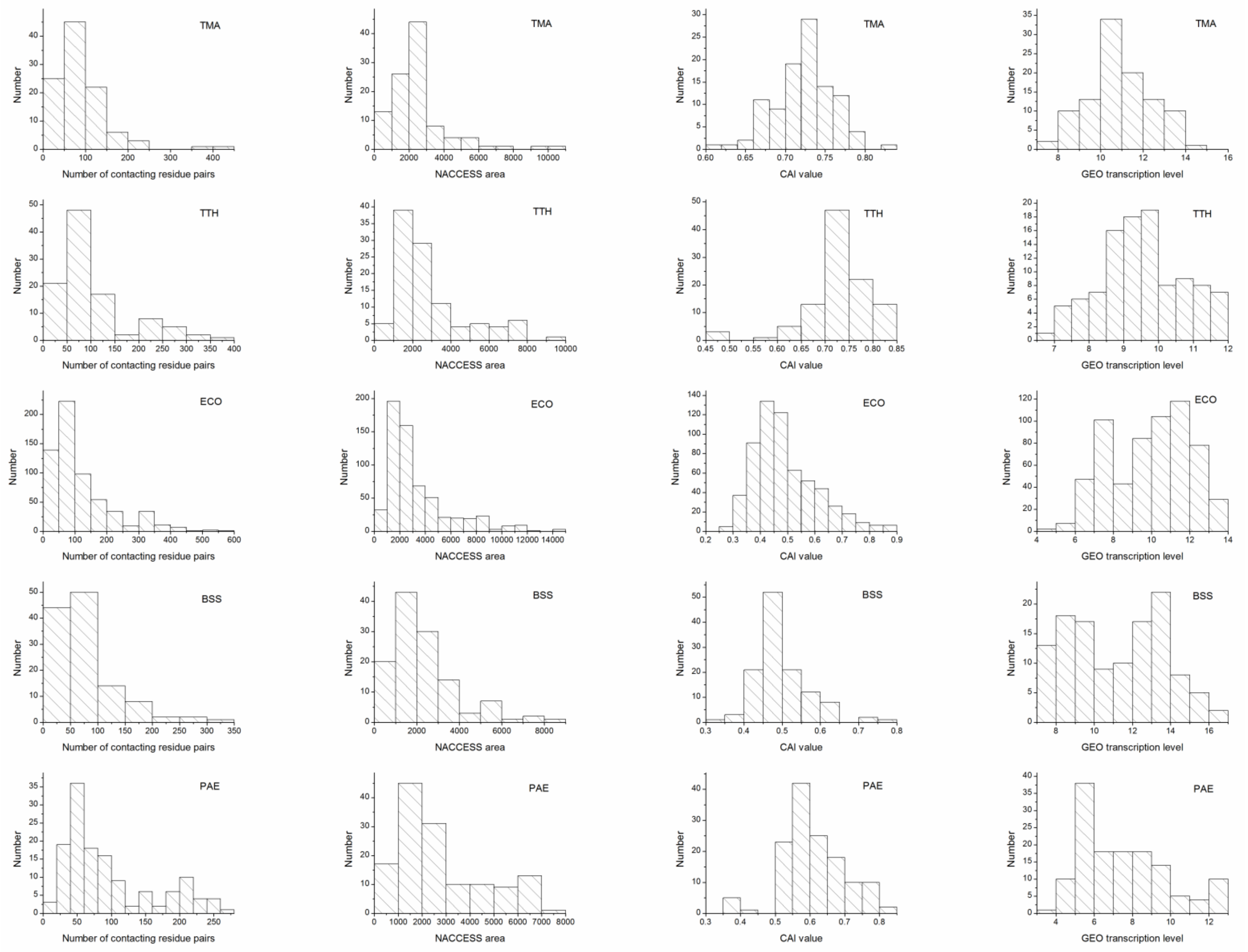

Figure S2. The distribution of the interface size and expression level for the protein complexes in the five studied organisms. Each row corresponds to an organism and each column corresponds to a quantity: interface size measured in the number of contacting residues (N_{aa}), interface size measured in the area calculated by NACCESS ($A_{naccess}$), expression level measured by CAI value, and expression level taken from GEO database, for the 1-4 column, respectively.

Supplementary Method: Simulation of protein interaction evolution by lattice model

1. The lattice model

A single chain biopolymer composed of 27 units can be put into a 3*3*3 lattice with each unit occupying a position in the lattice. The contacting matrix between the units in the lattice is defined as *C*: for two positions in the lattice, *m* and *n*, if the distance between *m* and *n* is just one unit, then *C* (*m*, *n*) = 1; otherwise, *C* (m, n) = 0. An example of the conformation on a lattice model is shown in **Fig. M1**.

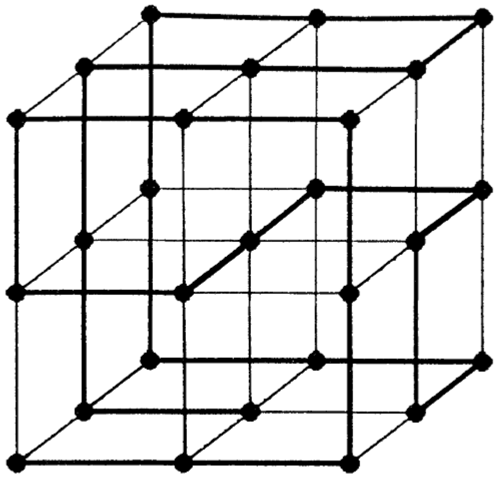

Fig. M1. An example of the conformation on a 3*3*3 cubic lattice (from the paper [1]).

For a specific conformation, its energy *E* can be represented as:

$$E = \frac{1}{2}\sum_{i,j}^{27} U(i,j)C(i,j)$$

where $U(i, j)$ is the contacting potential between the units *i* and *j* [1]. The 3*3*3 lattice model has sufficient complexity to give realistic insights as well as affordable computational burden to implement, and thus it is widely used in protein folding prediction, energy calculation and simulation of protein sequence evolution [2-5].

2. Amino acid interaction potential

In a peptide chain, we define the distance between two amino acid residues as the distance between their alpha carbon (C_α) atoms and deem the two residues contacting if this distance is less than 6.5 Å [6]. For example, the contact between amino acids *p* and *q* is defined according to the following formula:

$$\Delta_p^c \equiv \begin{cases} 0 & |p-q| \le 1 \\ \mathrm{H}\left(R^c - d_{pq}\right) & |p-q| > 1 \end{cases}, \quad R^c \equiv 6.5\text{Å}, \quad \mathrm{H}(x) \equiv \begin{cases} 1 & x \ge 0 \\ 0 & x < 0 \end{cases}.$$

For the residue *p*, its contacting potential is:

$$E_p^c(e_{ij}) = \frac{1}{2} \sum_{q(\neq p)} e_{i_p j_q} \Delta_{pq}^c ,$$

where the value of e_{ij} is calculated based on all the protein 3D structures in PDB as:

$$e^{-e_{ij}} = \frac{\bar{n}_{ij}\bar{n}_{00}}{\bar{n}_{i0}\bar{n}_{j0}} ,$$

in which n_{ij} is the contact number of residue types *i* and *j*, and 0 represents solvent molecules [6,7].

By calculation, we can get the contacting potential matrix between 20 types of amino acids; with this matrix, we can calculate the free energy of a protein structure or the binding energy between interacting proteins. A lot of amino acid contacting potential matrixes can be found in the AAindex database [8].

3. Determination of protein conformation

60 peptides of length 27 were randomly generated and folded into the 3*3*3 lattice model. The backtracking algorithm was used to enumerate all the 206692 non-repetitive folding conformations (if the symmetry of optical rotation is considered, there will be 103346 non-repetitive conformations). That is to say, starting from any of the 8 vertexes or the center point of any of the 6 faces of the cube, the next residue is searched on the 5 possible directions, and so on, until all the 27 residues are arranged on the 3*3*3 lattice. In the lattice model, each pair of residues with distance of 1 unit is treated as an internal interaction (namely contact) to stabilize the conformation. With the MJ matrix [7], the sum of the contacting potential energy of all the 54 contacting residue pairs for each conformation of each peptide chain is calculated and treated as the free energy for the specific conformation. Then, the F_i values for all the possible conformations of each protein sequence is calculated as:

$$F_i = \frac{E_i - M}{SD} ,$$

where E_i is the free energy of the *i*-th conformation for a protein, *M* is the mean value of the free energy for a protein over all the possible conformations, and *SD* is the standard deviation. For each protein sequence, we randomly selected one conformation from all the conformations with $F_i < -3.5$ as the native conformation for that protein. Therefore, 60 native conformations for the 60 proteins were determined and used in the subsequent simulation without further changes.

4. Determination of interacting protein pairs

In the lattice model, the interaction between two proteins with specific conformations are represented as a contacting interface composed of two faces of the 3*3*3 lattice cubes; therefore, the total number of the interaction forms between two proteins with specific conformations is 6*6*4. In the simulation, the interaction strength between two proteins with specific conformations is represented as the sum of the interaction strength values (binding energy) between the 9 corresponding contacting residue pairs on the two faces of the lattice cube. With the MJ matrix[7], the sum of the contacting potentials of the 9 pairs of contacting residues on the two contacting faces of the lattice cubes can be calculated and it is deemed as the binding energy

between the two interacting proteins in the two specific conformations.

For each protein, we calculated the binding energy between it and all of the other target proteins (including itself) according to the following formula:

$$B_{ijk} = F_{ijk} + F_{jik} , \qquad F_{ijk} = \frac{E_{ijk} - M_i}{SD_i} ,$$

where E_{ijk} is the binding energy between protein *i* and protein *j* in the *k*-th interaction (contacting) configuration, and M_i is the mean value of the binding energy between the *i*-th protein and all the other proteins in all the 8640 possible interaction (contacting) configurations, and SD_i is the corresponding standard deviation. All the contacting configurations with the $B_{ijk} < -3.2$ are treated as the possible interactions between the two proteins. The 60 proteins were appointed into 30 non-repetitive interacting pairs by using the Blossom algorithm to solve a maximum matching problem on general graphs. Finally, we got a set of interactions (interacting protein pairs) as:

$$S = \{(i, j) \mid \text{protein } i \text{ interacts with protein } j\} .$$

This set of interactions was used in the subsequent simulation without further variations.

5. Determination of protein expression level

In most species, the protein expression level keeps to a power-law distribution. In our simulation, 30 random numbers within the range of [1, 50] were generated according to a power-law distribution as the expression levels of the 30 interacting protein pairs and used in the simulation without further variations. The distribution of the randomly generated protein expression levels was shown in **Fig. M2**.

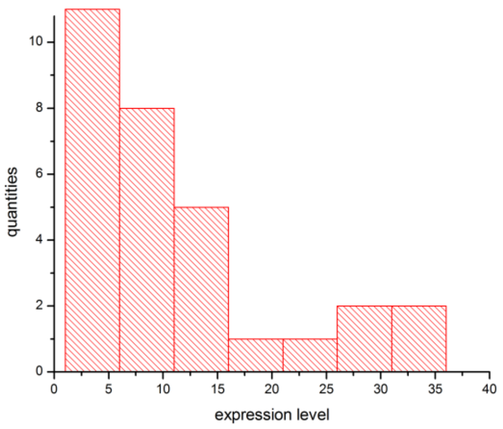

Fig. M2. The distribution of the randomly generated protein expression levels.

6. Cell fitness calculation

In the simulation, the 60 proteins were put into one system, called a cell. E_{ij} is the binding energy between protein *i* and protein *j*, which is determined by all the 144 possible interacting configurations and given by the following formula:

$$E_{ij} = -RT \ln \sum_{k=1}^{144} e^{-\frac{E_{ijk}}{RT}} ,$$

where R is the Boltzmann constant and T is the Kelvin temperature in the simulation.

Because the abundance of protein complexes is determined by both the stability of the binding interface and the temperature, we can get the abundance for each single protein unit and for the protein complexes in each cell by solving the following equations:

$$\frac{C_i C_j}{C_{ij}} = e^{\frac{E_{ij}}{RT}} \text{ and } D_i = C_i + \sum C_{ij} \ ,$$

where C_i and C_j are the abundance for the monomers protein i and protein j, respectively, and C_{ij} is the abundance for the protein complex formed by the interaction between protein i and protein j, and D_i is the expression level of the i-th protein. The above equation is a nonlinear equation with multiple variables; therefore, it was solved by numeric iteration according to the following formulae:

$$C_{n+1} = \frac{C_n D_i}{C_n + \sum C_{ij}} \ , \ d = \left| \sum C_{n+1} - \sum C_n \right| ,$$

until the difference between two successive iteration steps $d < 10^{-5}$.

Because the formation of stable protein complexes with specific functions is a necessity for the physiological function of a cell, we assume that the fitness of a cell is determined by the number of protein interactions in the proper configurations and thus define the fitness of a cell (denoted by A) as the sum of the abundance of the interacting proteins:

$$A = \sum_{(i,j) \in S} C_{ij} \ .$$

7. The simulation process

In the simulation process, 100 cells were generated as a population, with each cell containing 60 proteins. In the first generation, each cell has 60 proteins with initial settings. In the evolution process, for each generation, averagely 0.0005 mutations will occur for each amino acid in the protein sequences [9]. When mutation occurs, the original amino acid in the protein sequence will be replaced with another amino acid randomly selected from the other 19 residue types. In the evolution process, the protein abundance and the cell fitness were calculated after the finished mutations of each generation. Except for the cell population in the first generation, the cell population in each generation was formed by a random genetic drift from the previous generation. A simple random sampling strategy with replacement was adopted to generate the cell population of each generation from the previous generation: a cell was randomly sampled from the previous generation into the new generation, with the probability of being selected determined by its fitness, namely, the higher the fitness of a cell, the larger probability it was selected. The selection probability for cell i is Pi and calculated by the following formula:

$$P_i = \frac{A_i}{\sum A_i} \ ,$$

where A_i is the fitness value for cell i in the population. The random sampling procedure was repeated 100 times to get the new generation of cell population from the previous generation.

The whole evolution process simulated 10000 generations, and in each generation the fitness value

for each cell was calculated (**Fig. M3**).

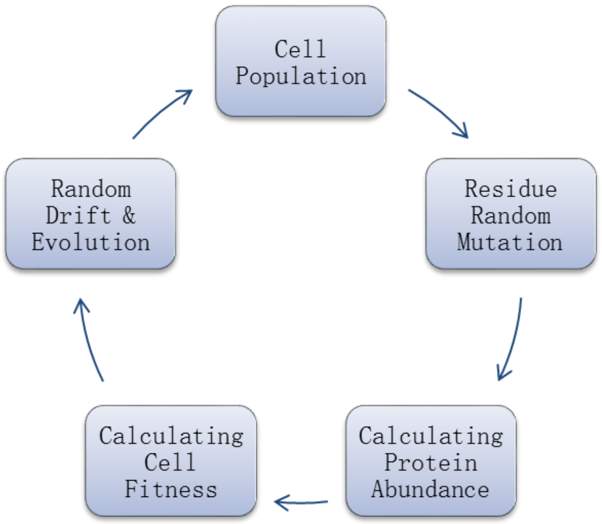

Fig. M3. The illustration for the simulation procedure of evolution.

Then, in each cell the binding energy (indicating the interface stability) for the interacting protein pairs of the top 10 expression levels and of the bottom 20 expression levels were calculated and compared at the high temperature (350K) and normal temperature (310K), respectively.

8. The change of cell population fitness at different temperature conditions

In the simulation process, the cell fitness values were calculated and averaged every 10 generations and plotted against the generation number in **Fig. M4** for high temperature and normal temperature, respectively.

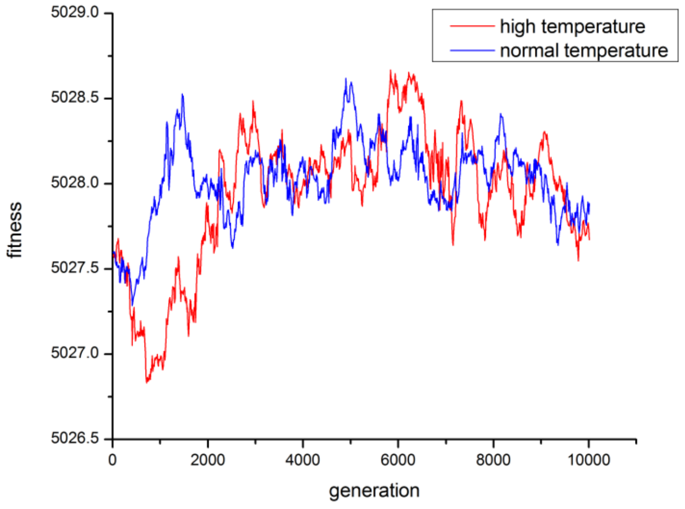

Fig. M4. The variations of cell fitness during the evolution process. The red line and the blue line indicate the average fitness of the cell populations evolving at high temperature (350K) and normal temperature (310K), respectively.

As can be seen, the fitness values for the cell populations increase rapidly at the early stage for

both the high and normal temperature conditions, and then the fitness values begin to fluctuate. The rapid increase of the fitness at the early stage is owing to the larger optimization space for the initial near random sequences that are more easily to mutate into more optimized sequences of better structure and interaction stability; with the progress of the optimization, the more optimized structures get less and less; therefore, although the overall trend for the evolution is still toward the higher fitness, the fluctuation of the fitness values is unavoidable due to the randomness of the mutations.

To avoid the effects of fluctuation on the subsequent analysis, the generation with the highest fitness (within 10000 generations) was selected as the terminal of the simulation; that is, the terminal for the high temperature condition is the 4900 generation while that for the normal temperature condition is 5840. The simulation is long enough to get meaningful results to reveal the different evolutionary strategies of protein interaction stability at different temperature conditions.

Table M1. The linear fitting for the fitness change

Temperature	Slope	R-square	p-value
High	6.83E-05	0.2420	<1E-16
Normal	1.95E-05	0.0562	1.83E-14

Linear fitting was applied for the fitness change and the results were summarized in **Table M1**. As shown, there are positive correlations between the fitness values and the generation numbers for both the high and normal temperatures, with the former having larger slope, meaning that cell population fitness increases more quickly at the high temperature condition than the low temperature condition, which results from the higher evolutionary pressure at the high temperature that preferentially select the cells with higher fitness (better stability).

9. The analysis of protein interaction interface stability at different temperature conditions.

The 30 protein pairs in each cell were partied into two groups: the top 10 pairs with higher expression levels (TOP) and the bottom 20 pairs with the lower expression levels (BTM), and the average binding energy over every 10 generations in the evolution was calculated and plotted against the generation number for the two groups, respectively. The evolution of the interface stability (the binding energy between interacting protein pairs) were simulated at the high temperature and normal temperature, respectively. For the simulation at the high temperature condition **(350K)**, as shown in **Fig. M5**, the binding energy values (shown as the absolute value, namely, the opposite number) increase (with fluctuation) during the evolution process for both the highly and lowly expressed proteins, meaning that the protein pairs evolve towards a direction of more stable interaction. Meanwhile, the increase speeds of the binding energy in the evolution that can be represented by the slope values of the linear fitting are different for the highly and lowly expressed protein pairs; namely, it is higher for the highly expressed proteins than the lowly expressed proteins.

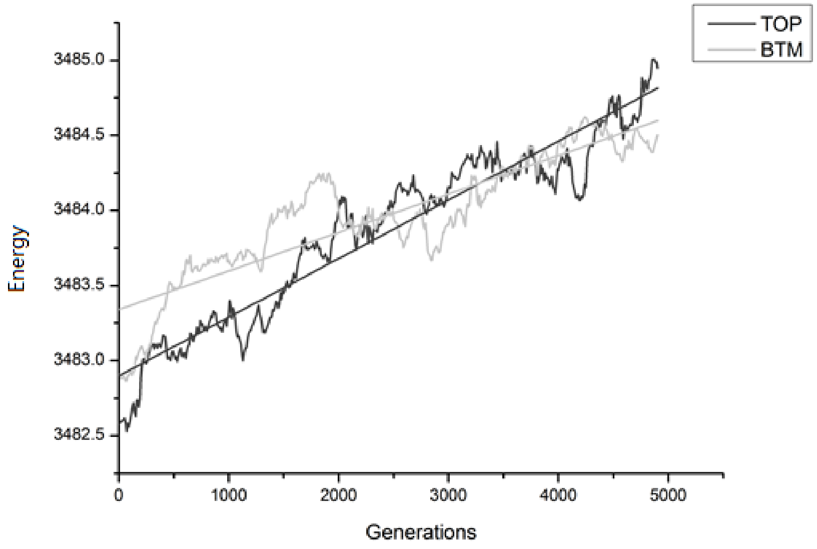

Fig. M5. The evolution of interface stability at high temperature (350K). The black lines indicate the binding energy of the protein complexes with the top 10 expression levels; the gray line indicates the binding energy of the protein complexes with the bottom 20 expression levels. Both the simulated data (broken lines) and the linear regression (straight lines) are shown.

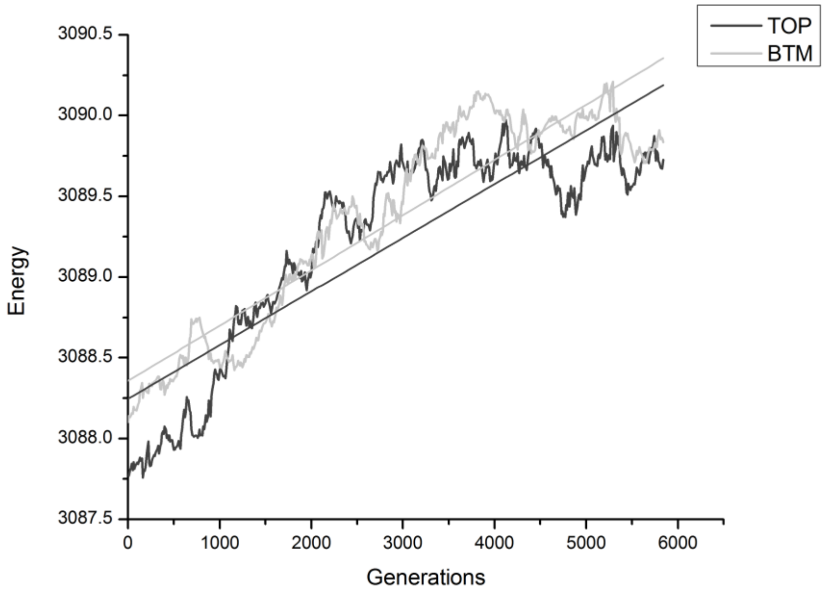

Fig. M6. The evolution of interface stability at normal temperature (310K). The black lines indicate the binding energy of the protein complexes with the top 10 expression levels; the gray lines indicate the binding energy of the protein complexes with the bottom 20 expression levels. Both the simulated data (broken lines) and the linear regression (straight lines) are shown.

Linear fitting was applied to the curves in **Fig. M5** and the corresponding parameters were presented in **Table 4**. The slope of the fitted line represents the evolution speed of the interface stability (binding energy). At the high temperature condition, it can be found that the slope of the fitted line for highly expressed protein pairs is 3.91E-04 while that for the lowly expressed protein pairs is 2.56E-04 (**Table 4**); the former is larger than the latter, meaning that the interface stability of the interacting protein pairs evolves faster for the highly expressed protein pairs. Similar analysis was performed for the protein interface evolution at the normal temperature **(310K)** and the results were presented in **Fig. M6** and **Table 4**. It can be found that the slope of the fitted line for the highly expressed protein pairs (3.32E-04) is very close to that for the lowly expressed protein pairs (3.42E-04), meaning that the evolution speeds of the interface stability for the highly and lowly expressed protein pairs are similar to each other at the normal temperature condition.

References

[1] Shakhnovich, E. and Gutin, A.M. (1990). Enumeration of all compact conformations of copolymers with random sequence of links. The Journal of Chemical Physics 93, 5967-71.

[2] Shakhnovich, E., Farztdinov, G., Gutin, A.M. and Karplus, M. (1991). Protein folding bottlenecks: A lattice Monte Carlo simulation. Phys Rev Lett 67, 1665-1668.

[3] Mirny, L.A. and Shakhnovich, E.I. (1996). How to derive a protein folding potential? A new approach to an old problem. J Mol Biol 264, 1164-79.

[4] Berezovsky, I.N., Zeldovich, K.B. and Shakhnovich, E.I. (2007). Positive and negative design in stability and thermal adaptation of natural proteins. PLoS Comput Biol 3, e52.

[5] Deeds, E.J., Ashenberg, O., Gerardin, J. and Shakhnovich, E.I. (2007). Robust protein protein interactions in crowded cellular environments. Proc Natl Acad Sci U S A 104, 14952-7.

[6] Miyazawa, S. and Jernigan, R.L. (1985). Estimation of Effective Interresidue Contact Energies from Protein Crystal Structures: Quasi-Chemical Approximation. Macromolecules 18, 534-52.

[7] Miyazawa, S. and Jernigan, R.L. (1996). Residue-residue potentials with a favorable contact pair term and an unfavorable high packing density term, for simulation and threading. J Mol Biol 256, 623-44.

[8] Kawashima, S., Pokarowski, P., Pokarowska, M., Kolinski, A., Katayama, T. and Kanehisa, M. (2008). AAindex: amino acid index database, progress report 2008. Nucleic Acids Res 36, D202-5.

[9] Yang, J.R., Liao, B.Y., Zhuang, S.M. and Zhang, J. (2012). Protein misinteraction avoidance causes highly expressed proteins to evolve slowly. Proc Natl Acad Sci U S A 109, E831-40.
